# Methods for integrating postural control into biomechanical human simulations: a systematic review

**DOI:** 10.1186/s12984-023-01235-3

**Published:** 2023-08-21

**Authors:** Julian Shanbhag, Alexander Wolf, Iris Wechsler, Sophie Fleischmann, Jürgen Winkler, Sigrid Leyendecker, Bjoern M. Eskofier, Anne D. Koelewijn, Sandro Wartzack, Jörg Miehling

**Affiliations:** 1 Engineering Design, Department of Mechanical Engineering, Friedrich-Alexander-Universität Erlangen-Nürnberg, Erlangen, Germany; 2 Machine Learning and Data Analytics Lab, Department Artificial Intelligence in Biomedical Engineering (AIBE), Friedrich-Alexander-Universität Erlangen-Nürnberg, Erlangen, Germany; 3 Department of Molecular Neurology, University Hospital Erlangen, Friedrich-Alexander-Universität Erlangen-Nürnberg, Erlangen, Germany; 4 Institute of Applied Dynamics, Department of Mechanical Engineering, Friedrich-Alexander-Universität Erlangen-Nürnberg, Erlangen, Germany

**Keywords:** Systematic review, Postural control, Biomechanics, Biomechanical human model, Musculoskeletal model, Forward dynamics, Simulation

## Abstract

Understanding of the human body’s internal processes to maintain balance is fundamental to simulate postural control behaviour. The body uses multiple sensory systems’ information to obtain a reliable estimate about the current body state. This information is used to control the reactive behaviour to maintain balance. To predict a certain motion behaviour with knowledge of the muscle forces, forward dynamic simulations of biomechanical human models can be utilized. We aim to use predictive postural control simulations to give therapy recommendations to patients suffering from postural disorders in the future. It is important to know which types of modelling approaches already exist to apply such predictive forward dynamic simulations. Current literature provides different models that aim to simulate human postural control. We conducted a systematic literature research to identify the different approaches of postural control models. The different approaches are discussed regarding their applied biomechanical models, sensory representation, sensory integration, and control methods in standing and gait simulations. We searched on Scopus, Web of Science and PubMed using a search string, scanned 1253 records, and found 102 studies to be eligible for inclusion. The included studies use different ways for sensory representation and integration, although underlying neural processes still remain unclear. We found that for postural control optimal control methods like linear quadratic regulators and model predictive control methods are used less, when models’ level of details is increasing, and nonlinearities become more important. Considering musculoskeletal models, reflex-based and PD controllers are mainly applied and show promising results, as they aim to create human-like motion behaviour considering physiological processes.

## Introduction

### Motivation

During standing and gait, the human body is an inherently unstable biomechanical system. Keeping balance during standing and gait is a complex task controlled by the central nervous system (CNS), consisting of low-level controls generated in the spinal cord as well as higher-level controls generated in the supraspinal cord. This process is called postural control. Simulating postural control behaviour requires a fundamental understanding of the human body’s internal processes that are responsible for balancing. We would like to use postural control simulations to give therapy recommendations to patients suffering from postural control disorders in the future, by suggesting targeted exercises for restoration of body functions or to estimate the influence of specific exercises. This is possible when these simulations behave similarly to real postural control. Current literature provides different models that aim to simulate the mechanisms of the body to keep balance when standing, during gait, or while performing other tasks. These control methods are applied to biomechanical human models to understand the interaction between the dynamics and the control of the model. Depending on the scope, biomechanical human models can be highly simplified to simulate the general motion behaviour or very detailed to focus on the internal processes during postural control as well. Current approaches range from 1 degree of freedom (DoF) two-dimensional single inverted pendulum models [75], to more DoF in double [23] or triple inverted pendulum models [28], up to complex three-dimensional musculoskeletal models [86]. Depending on the model detail, the focus is on a general, specific, or extensive understanding and simulation of motion behaviour. Such a simulation should include the different sensory systems that the body uses to sense its internal states and body dynamics. However, until today it is not fully understood how the human body processes this sensory information in the CNS to react adequately to maintain balance, in perturbed as well as in unperturbed situations [35, 44, 95].

To predict a certain motion behaviour, forward dynamic simulations can be used with joint moments or muscle forces as input. Forward dynamic simulations lead from specific joint torques or muscle activations to a motion synthesis without the need of further experimental motion data. This allows for investigating correlations between control inputs, model dynamics and performed tasks [108]. Such a simulation could aim a balancing task in standing scenarios, where the centre of mass (CoM) should maintain within the base of support, which is described by the area within the contact points of the feet with the ground. Such a simulation could also aim generating a stable gait in walking scenarios that is robust against external perturbations, by combining predefined gait patterns combined with controlling, reactive elements. To apply predictive forward dynamic simulations for our own purposes, it is important to know which modelling approaches already exist, when they are applied preferably, what the scope of their application in research is and how can this modelling approach can be implemented in a postural control model to generate human motion behaviour as naturally as possible. Furthermore, it is important that sensory systems and control methods are represented accurately when using the simulations for therapy recommendations in the future.

Digital human models are also used for motion control in other fields, such as character animation. In Cruz Ruiz et al. [16], for example, different simulation strategies from this research area are compared. Therefore, we focus on the application area of biomechanics in the following.

In this paper, we give an overview about previously published models that are simulating the human’s postural control behaviour including sensory feedback of the body. We thereby answer the following research questions conducting a systematic literature research:

- RQ1: What kinds of biomechanical human models are used to simulate the body’s kinematics and dynamics?
- RQ2: How are the body’s sensory systems represented by existing models and how are their sensory information integrated into the postural control model?
- RQ3: Which control methods can be used to implement postural control mechanisms in biomechanical human models?

### Biological background

In the following section, the biological background for postural control simulations is described. There are many different terms to describe human behaviour or reactions to maintain an erect position during static or dynamic situations. The CNS regulates the sensory information from different receptors of the body to initiate adequate muscle reactions. Faster, lower-level controls, such as reflexes, are generated in the spinal cord, higher-level controls are generated in the supra-spinal cord. Postural control, balance control, or balance behaviour are terms used to describe this process to maintain balance. In the following we will use the phrase postural control.

**Fig. 1:**
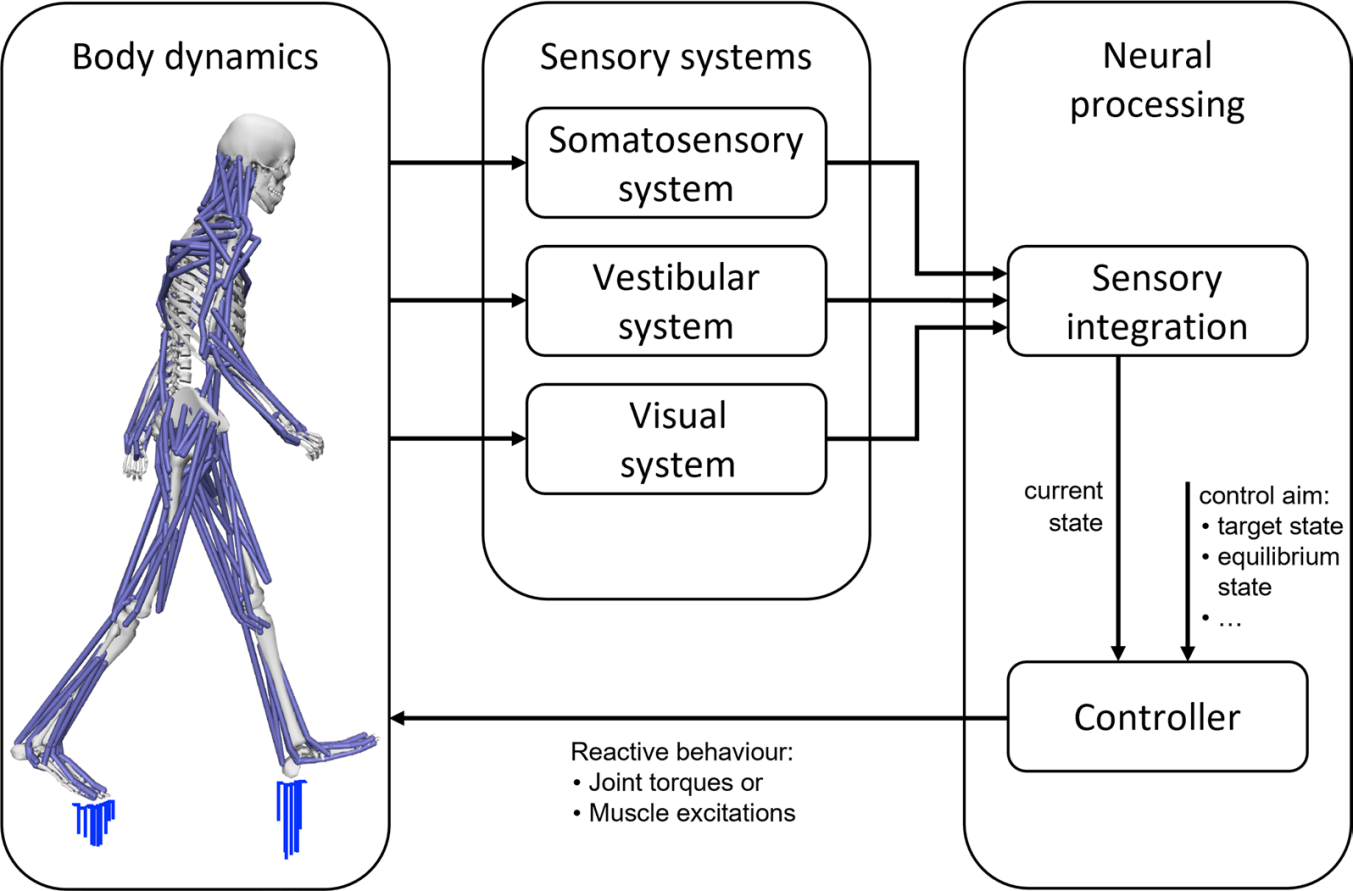
Simplified control system model. Body dynamics can be observed by several sensory systems of the body. This gained information has to be integrated to obtain an accurate body perception. The controller uses the current states and a specific control aim, like target states or equilibrium states, to determine the subsequent movements of the body

To maintain balance, the human body uses the information of several receptor types, like muscle length or the pressure distribution underneath the feet, to observe the internal states of the body kinematics and dynamics. Relevant components in this respect are the somatosensory, vestibular, and visual systems [15, 41]. The information of all these sensory systems is interpreted in the CNS to initiate muscle excitations, which translates into adequate muscle forces to keep the body in balance. Figure 1 shows a general overview of the postural control processes. The somatosensory system consists of proprioception and cutaneous receptors. Through proprioception, the body gains information about the relative orientation and movements of body segments. Muscle lengths and velocities are sensed by muscle spindles located in the skeletal muscles, joint angles by joint receptors located in the joint capsules. Tendon forces are sensed by Golgi tendon organs located at the interface between muscles and tendons. Cutaneous receptors on the plantar feet surface deliver information about pressure distribution. Additionally, from this delivered information the body can estimate the geometry of the support surface [41]. The vestibular system senses linear and angular motion and the orientation of the head with respect to gravity. This sensory system is located within the inner ear and consists of two otolith organs and the semi-circular canals. The otolith organs detect linear accelerations of the head, the semi-circular canals identify angular head accelerations [41]. Visual feedback is used to get information about the orientation and motion of the head and the whole body with respect to its environment. The CNS can use this information to interpret the direction and speed of body sway, for example. It has to be mentioned that visual feedback alone can be interpreted ambiguously, either as self-motion of the head or body, or as motion of the surrounding objects [41].

Using the previously described different sensory systems, the body gains channel-specific information, depending on the particular sensory system, about the body kinematics and dynamics. Every single channel suffers from noise and delivered values can differ from each other. Therefore, it is necessary that the feedback information is integrated in the CNS to provide stable and accurate estimates about the body kinematics and dynamics. A reliable perception of current body states is important to generate suitable postural control reactions [41]. In order to adapt to changing environmental conditions or a disturbance of a sensory system, the body is able to reweigh the relative contributions of the sensory channels [58, 67, 74].

The body applies different ways to keep balance using previously processed sensory information. It is necessary to maintain the CoM inside the base of support to ensure a stable posture. During standing, this can be achieved for example by correcting mechanisms in the ankle or hip [1, 37]. These correcting mechanisms shift the centre of pressure (CoP), and thereby control the movement of the CoM. When these corrections are no longer sufficient, humans can apply further movements, like one or more recovery steps. During gait, more dynamic stability approaches are used, as the CoM is not necessarily always inside the base of support. Mechanisms like foot placement of the next stance leg and hip torque control of the current stance leg are important to stabilize the trunk during gait [85]. Additionally, in case of a perturbation, recovery behaviour like adapted step length and step time can be identified [45].

These methods that are applied to maintain balance can be split into feedback and feedforward mechanisms. Feedback means that current sensory information is included to generate appropriate reactions, as the body does with postural control. Feedforward means that voluntary motor control is performed in a pre-defined way. The contribution of feedforward elements in human’s postural control are still being discussed in literature [19, 29, 87].

The time delay represents another physiologically relevant parameter for the resulting reaction time on perturbations. An information transfer of sensory information through afferent and signals for muscle control through efferent pathways, as well as information processing on spinal and supra-spinal levels in the CNS, lead to time delays in postural control. This process is continuously present and therefore important for the standing as well as gait movements. Thus, time delay represents an important parameter for modelling as well and has an effect on both feedback and feedforward elements.

## Methods

**Fig. 2:**
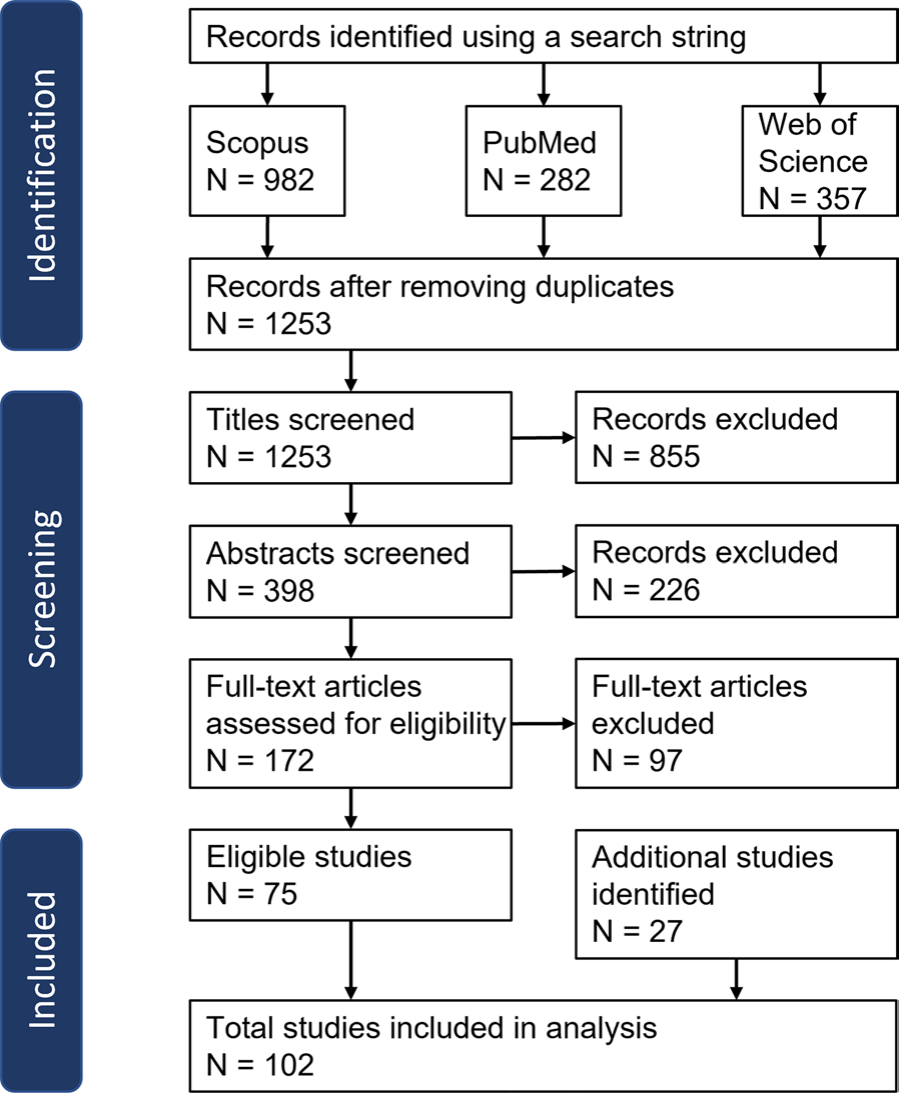
Systematic protocol of identification and scanning of literature. A search string was used to identify records from Scopus, PubMed, and Web of Science. After removing duplicates, records were screened and filtered by inclusion criteria. 102 studies have been included to be further analysed in this review

We followed a systematic protocol in order to identify significant literature regarding the previously mentioned research questions (Fig. 2). We applied a search string to ensure a transparent, reproducible procedure and screened the resulting records’ content manually afterwards. The search string consisted of elements identifying biomechanical human models that are describing postural control during standing and gait simulations:

[(musculoskel*) OR (musculo-skel*) OR (biomechani*) OR (multisegment*) OR (multi-segment*) OR (neuromuscul*) OR (multi-joint) OR (multijoint) OR ("inverted pendulum")]

AND [human*]

AND [(simulation*) OR (modulation*) OR (numeric) OR (model*)]

AND [(postur*) OR (balanc*) OR (stabili*) OR (reflex*) OR (reacti*) OR (equilibrium) OR (propriocept*) OR (sensor*) OR (feedback)]

W/2 [(control*) OR (behavio?r) OR (recover*)]

AND [(stance) OR (standing) OR (gait) OR (walking) OR (sway)]

The search was performed using three different databases: Scopus, PubMed, and Web of Science. In Scopus, we applied the search for *title, abstract and keywords* and limited the outcomes to the document type *article* and subject areas *engineering*, *computer science*, *mathematics*, *medicine*, *multidisciplinary*, *neuroscience* and *physics and astronomy*. As available search criteria differ between Scopus, PubMed and Web of Science, we had to adapt the search in PubMed and Web of Science: In PubMed, we applied the search string to *title/abstract* and limited the outcomes to the publication type *article* as well, English language and the filter criterion *humans*. In Web of Science (Core collection), we applied the search string to *title, abstract, keywords plus* where we limited the results to English language and the document type *articles*. Applying the search string to PubMed and Web of Science, we had to replace the operator *W/2* by *AND*. We identified studies which fulfilled the following inclusion criteria: the studies contain a postural control model of the human that describes the sensory feedback dynamics to regulate the postural control; the model has to be new or a significant extension of an existing model; and the model should be usable for forward dynamic simulations. The focus of this review is on models used for standing or gait conditions, considering the upper and lower body (simplifications were accepted). The model has to simulate at least the dynamic and control behaviour during physiological motion of standing or gait. As this review aims to give an overview about models used for simulations of human movement behaviour, applications with focus on humanoids, exoskeletons, or prosthetics are excluded.

The literature that we found with the search string was screened next considering the previously mentioned inclusion criteria, first by their titles and abstracts, then as full-texts. Since many different terms are used to describe the human’s postural control in literature, we are aware that a search string cannot find all matching publications. To compensate for that, we additionally included a backward search by scanning the references of the included studies regarding our inclusion and exclusion criteria.

The included studies were analysed and categorized in the following aspects:

- What kind of biomechanical model is considered to describe the body’s dynamics?
- How are the body’s sensory systems for postural control integrated into the models?
- What kind of control method is implemented to process sensory information?

Including all publications up to April 28th, 2023, we identified 1253 publications with the search string. After screening according to our protocol, which is summarized in Fig. 2, we included 102 studies.

## Results

### Biomechanical models

We identified several different approaches for modelling and simulating human postural control. In these approaches, the level of detail of the biomechanical human models varies, since this level is adjusted to the scope and research questions that are identified. In general, applied models vary between non-musculoskeletal and musculoskeletal models and are used for either standing or gait simulations. Musculoskeletal models add complexity through a larger number of DoF and by including muscle dynamics, joints can be modelled closely to the anatomy [18]. Actuation of non-musculoskeletal models can be realized by joint torques, actuation of musculoskeletal models by muscle excitations. In the following we give an overview about biomechanical human models used for postural control simulations. We clustered biomechanical human models into non-musculoskeletal and musculoskeletal models, each during standing and gait simulations. Tables 1 and 2 show non-musculoskeletal (Table 1) and musculoskeletal models (Table 2) during standing simulations, Tables 3 and 4 non-musculoskeletal (Table 3) and musculoskeletal models (Table 4) during gait simulations.

**Table 1:** Classification of the included studies by the applied biomechanical human model during standing simulations: non-musculoskeletal models during standing simulations

1 Joint			2 Joints		3 Joints		4 Joints		5 Joints	
1 DoF	[3]	[67]	2 DoF	[10]	3 DoF	[4]	1 DoF	[9]	5 DoF	[38]
	[7]	[68]		[11]		[28]				
	[12]	[69]		[23]		[40]				
	[20]	[71]		[26]		[60]				
	[25]	[75]		[33]		[96]				
	[34]	[74]		[36]		[97]				
	[39]	[77]		[35]						
	[48]	[80]		[44]						
	[52]	[82]		[53]						
	[57]	[98]		[54]						
	[63]	[102]		[58]						
	[64]	[104]		[61]						
	[65]	[110]		[62]						
	[66]			[76]*						
				[89]						
2 DoF	[2]			[91]						
	[13]*									
	[14]*		4 DoF	[21]*						
	[105]*									
		∑ = 31		∑ = 17		∑ = 6		∑ = 1		∑ = 1

Models are classified by their number of represented joints as well as internal degrees of freedom (DoF)

*The motion of the model is considered three-dimensionally

**Table 2:** Classification of the included studies by the applied biomechanical human model during standing simulations: musculoskeletal models during standing simulations

1 Joint		3 Joints		6 Joints		7 Joints		8 Joints	
1 DoF	[99]	3 DoF	[46]	6 DoF	[90]	7 DoF	[42]	12 DoF	[101]*
	[100]				[56]		[43]		
	[107]								
						15 DoF	[50]*		
	∑ = 3		∑ = 1		∑ = 2		∑ = 3		∑ = 1

Models are classified by their number of represented joints as well as internal degrees of freedom (DoF)

*The motion of the model is considered three-dimensionally

**Table 3:** Classification of the included studies by the applied biomechanical human model during gait simulations: non-musculoskeletal models during gait simulations

2 Joints		4 Joints		6 Joints		8 Joints	
2 DoF	[85]	4 DoF	[94]	6 DoF	[22]	8 DoF	[70]
			[93]		[106]		
3 DoF	[8]*						
	[59]*						
4 DoF	[49]*						
	∑ = 4		∑ = 2		∑ = 2		∑ = 1

Models are classified by their number of represented joints as well as internal degrees of freedom (DoF)

*The motion of the model is considered three-dimensionally

**Table 4:** Classification of the included studies by the applied biomechanical human model during gait simulations: musculoskeletal models during gait simulations

2 Joints		6 Joints		7 Joints		10 Joints		11 Joints		13 Joints	
2 DoF	[17]	6 DoF	[6]	7 DoF	[92]	10 DoF	[27]	17 DoF	[109]*	19 DoF	[32]*
			[5]								[31]*
			[19]								
			[24]	17 DoF	[84]*					23 DoF	[55﻿]
			[29]								
			[30]								
			[45]								
			[47]								
			[72]								
			[73]								
			[81]								
			[88]								
			[95]								
		8 DoF	[51]*								
			[78]								
			[79]								
			[86]*								
			[87]*								
			[103]*								
	∑ = 1		∑ = 19		∑ = 2		∑ = 1		∑ = 1		∑ = 3

Models are classified by their number of represented joints as well as internal degrees of freedom (DoF)

*The motion of the model is considered three-dimensionally

66 of 102 papers describe standing simulations. 56 times non-musculoskeletal, 10 times musculoskeletal standing models are used. We divided the standing models into groups depending on the number of joints as well as DoF. To ensure comparability, DoF are represented as internal DoF in this paper. Models may contain additional DoF to the global frame, like relative rotations or translations with respect to the ground. Non-musculoskeletal standing models are divided into 1-joint models [2, 100, 102] up to 5-joint models [38], musculoskeletal standing models into 1-joint [107] up to 8-joint models [101]. Standing models focusing on the sagittal plane often merge left and right segments: 1-joint models describe the motion around the (merged) ankle joint and are comparable to single inverted pendulums [7, 74, 105]. Even if no foot is modelled, this joint can be considered as ankle joint during standing. 2-joint models describe the motion around the ankle and hip joint and are comparable to double inverted pendulums [53, 58, 62]. 3-joint models describe the motion around the ankle, knee and hip joint and are comparable to triple inverted pendulums [4, 28, 97]. Included musculoskeletal models using more than 3 joints consider left and right segments separately [42, 90, 101]. More complex standing models include additional joints like a lumbar joint [42, 50], subtalar joints [101] or shoulder and elbow joints [38]. The number of DoF depends from the number of modelled joints and whether the model is implemented two-dimensionally or three-dimensionally.

36 of 102 papers describe gait simulations. Non-musculoskeletal models are used 9 times, musculoskeletal models 27 times. Again, we divided the models into groups depending on the number of joints. Non-musculoskeletal gait models are divided into 2-joint models [8, 49, 59] up to a 8-joint model [70], musculoskeletal models into 2-joint models [17] over 6-joint models [6, 24, 29] up to 13-joint models [31, 32, 55]. When simulating gait, models consider left and right segments separately. 2-joint gait models describe the motion around the hip joints without considering a separate foot segment, 4-joint models describe motion around ankle and hip joints, while 6 joint-models also include motion around the knee joint. More complex gait models include additional joints like a lumbar joint [92] or shoulder and elbow joints [31, 55].

### Sensory representation and integration methods

The implemented representation of the body’s sensory systems shows to be very diverse. The sensory input that is fed into the control model varies depending on the used biomechanical model, its DoF and the research questions of the respective studies. Some authors do not specify which sensory systems they aimed to replicate in their postural control models. Often, full-state information from different segments, such as joint angles, angular velocities and sometimes angular accelerations are used [104]. Another frequently used parameter is the CoM [85, 101, 105]. If the research focuses on reproducing the body’s sensory systems and their measured information in a way similar to biology, the different senses of the postural control system are included.

Mostly, the somatosensory system and therefore proprioceptive and cutaneous receptors’ information is implemented. Muscle spindles have been imitated by tracking joint angles and angular velocities in more abstract biomechanical models [80, 97] and muscle length and velocity feedback in musculoskeletal models [24, 43, 56]. Golgi tendon organs have been simulated by torque [46, 80], or muscle force feedback equivalently [24, 56]. The information that the body receives from cutaneous receptors on the plantar feet surface can be modelled using the shift of the CoM’s gravitational vector [68] or by calculating the ground reaction forces. Cutaneous receptors provide information about the motion of the body with respect to the support surface [74].

Though most studies do not include a model of the biological sensing system, or only consider a model of the somatosensory system, vestibular feedback is sometimes taken into account as well. The semi-circular canals can be modelled using the rotational acceleration of the head in the world frame, while the otolith organs can be modelled using the translational acceleration of the head in the world frame [43, 52, 97]. In more simplified models, global trunk angles and angular velocities are interpreted as vestibular information as well [25, 36].

Additionally, some studies include visual information. The visual system can be estimated using the global position and velocity of the head [25, 74].

If sensory information from multiple different channels is used, this information must be fused and integrated to obtain a stable and reliable estimate of the actual body dynamics. The CNS provides this mechanism in the body. The benefit of this redundancy of information is that perturbed or erroneous sensory information can be detected and a more robust interpretation of the current situation is possible. This redundancy leads to the ability of the body to compensate even for the absence of some sensory information to a certain degree and still to be able to maintain balance. Sensory integration and reweighing mechanisms are applied trying to replicate the CNS’ processes in the different models. In this review we use the classification of Maurer et al. [67], who cluster sensory integration methods into three types: models that simply add the different sensory information without taking dependencies into account, models that use optimal estimator methods for adaptive sensory reweighing and models that allow interactions between the different information paths. The first method is introduced as *independent channel model* [74]. A model that includes optimal estimators that reweigh the different sensory information is called *optimal estimator model*. Applying this method, Van Der Kooij et al. [97, 98] use a Kalman filter for sensory reweighing. The third method is called *intersensory interaction model*. It is in general comparable to the independent channel model, but additionally allows for interactions between the several sensory channels [68]. Table 5 gives an overview about the different sensory integration methods used by the studies included in this review. The studies listed in this table use multiple sensory inputs for their postural control models. Only few studies considering multiple sensor types use musculoskeletal models [43, 87, 107] or gait simulations [45, 51, 87]. This processed sensory information serves as input for the applied control methods, which are addressed in Sect. “Postural control methods”.

**Table 5:** Sensory integration methods

Independent channel models	Optimal estimator models	Intersensory interaction models
***Standing models***		
[23]	[58]	[36]
[25]	[97]	[35]
[26]	[98]	[63]
[43]^m^		[67]
[52]		[68]
[64]		[82]
[74]		
[99]^m^		
[107]^m^		
***Gait models***		
		[45]^m^
		[51]^m^
		[78]^m^
		[79]^m^
		[87]^m^

Included studies considering more than one sensory system of the body are classified by the different applied methods to integrate the different sensory information (using the classification of Maurer et al. [67])

^m^The study uses a musculoskeletal model

### Postural control methods

**Fig. 3:**
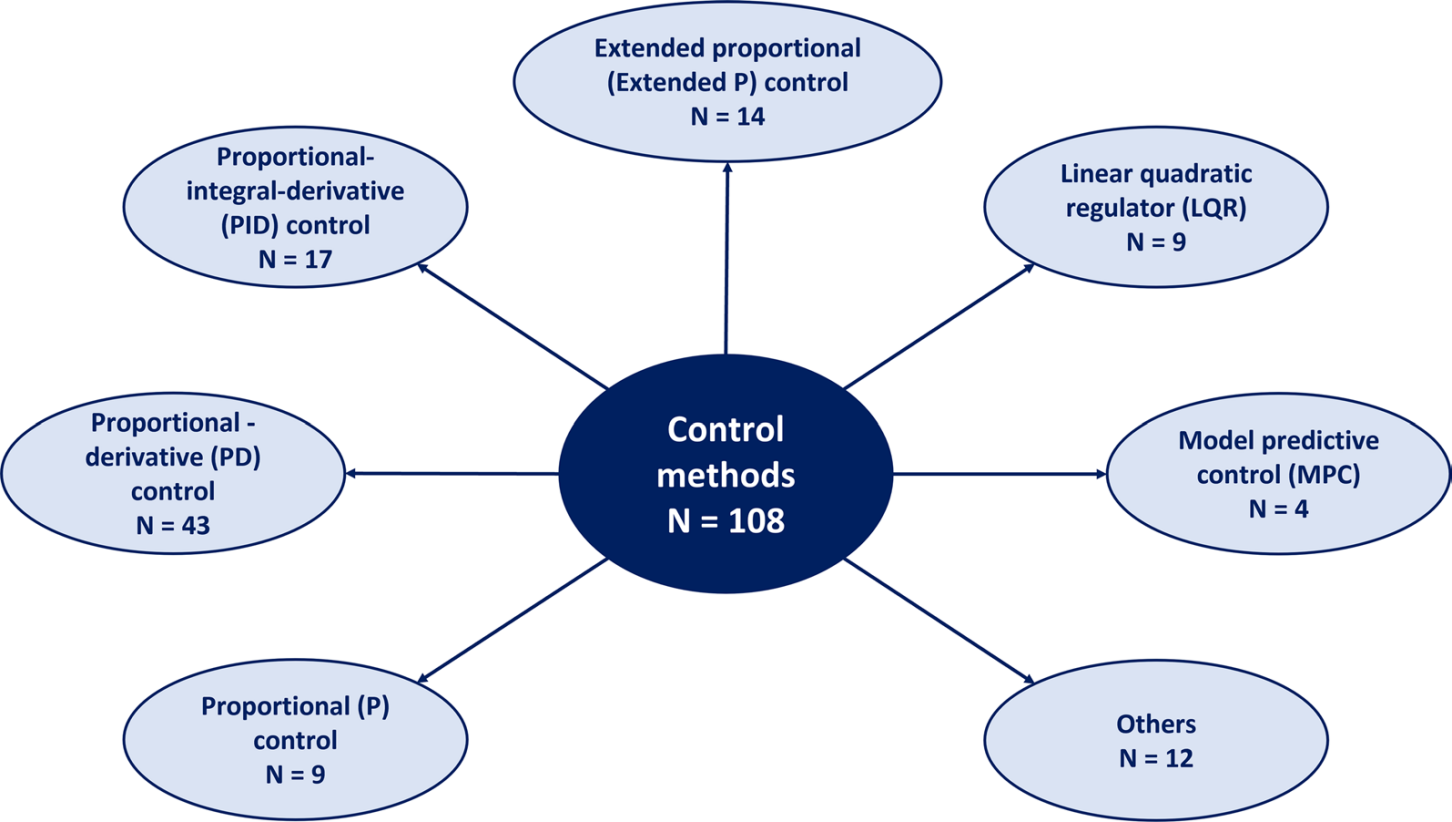
Classification of postural control methods. Scanning the 102 included studies a total of 108 control methods is applied (6 studies did apply a combination of two methods). Proportional (P) controllers are used in 9, proportional-derivative (PD) control in 43, proportional-integral-derivative (PID) control in 17 models. Extended P controllers are considered 14 times. Authors of 9 studies applied linear quadratic regulators (LQRs), of 4 studies model predictive control (MPC). In models of 12 studies other approaches are used

**Fig. 4:**
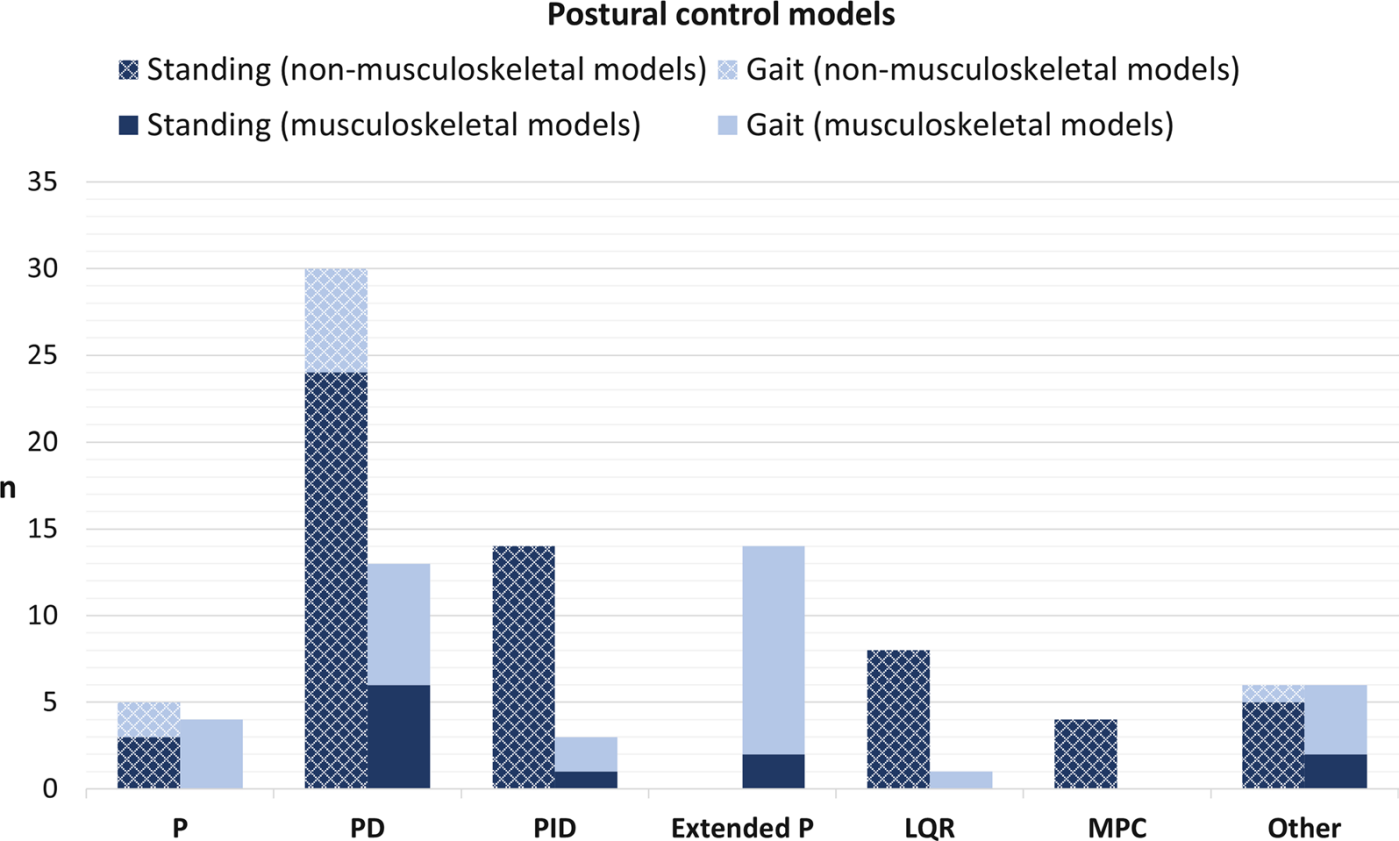
Postural control models that have been used by the included studies. They are classified by the control method and within these categories separated by non-musculoskeletal (left column each) and musculoskeletal models (right column each), as well as by standing and gait applications

We classified the studies according to their different control methods applied to the postural control models. This classification gives an impression of the diversity of considered control methods in postural control models (Fig. 3) and which control methods is used in combination with which type of biomechanical human model (Fig. 4). The 102 included studies show a total of 108 control approaches, 6 studies did apply a combination of two methods. We identified 6 different types of control methods: Proportional (P), proportional-derivative (PD), proportional-integral-derivative (PID) controllers, reflex-based controllers, linear quadratic regulators (LQR) and model predictive control (MPC). Table 6 shows all included studies and their classification into the different control methods. Following we give an overview about the different identified control methods.

**Table 6:** Classification of the included studies by the applied postural control methods, separated into standing and gait models

P controller	PD controller	PID controller	Extended P controller	Linear quadratic regulator (LQR)	Model predictive control (MPC)	Others
***Standing models***						
[12]**°**	[4]	[13]	[56]^m^	[53]	[3]	[40]
[23]	[7]	[14]	[90]^m^	[60]	[2]	[52]°
[28]	[9]	[25]		[58]	[38]	[71]
	[10]	[36]		[61]	[62]	[101]^m^
	[11]	[39]		[77]		[104]
	[12]**°**	[44]		[76]		[105]
	[20]	[46]^m^		[96]		[107]^m^ °
	[21]	[48]		[97]		
	[26]	[64]				
	[34]	[67]				
	[33]	[68]				
	[35]	[75]				
	[42]^m^	[74]				
	[43]^m^	[80]				
	[50]^m^	[82]				
	[52]°					
	[54]					
	[57]					
	[63]					
	[65]					
	[66]					
	[69]					
	[89]					
	[91]					
	[98]					
	[99]^m^					
	[100]^m^					
	[102]					
	[107]^m^ °					
	[110]					
∑ = 3	∑ = 30	∑ = 15	∑ = 2	∑ = 8	∑ = 4	∑ = 7
***Gait models***						
[5]^m^ °	[6]^m^	[47]^m^	[19]^m^	[17]^m^ °		[32]^m^
[8]	[5]^m^ °	[84]^m^	[24]^m^			[31]^m^
[17]^m^ °	[22]		[29]^m^			[55]^m^
[72]^m^	[27]^m^		[30]^m^			[59]
[85]	[45]^m^		[51]^m^			[109]^m^
[95]^m^ °	[49]		[78]^m^			
	[70]		[79]^m^			
	[73]^m^		[81]^m^			
	[94]		[88]^m^			
	[93]		[86]^m^			
	[92]^m^		[87]^m^			
	[95]^m^ °		[103]^m^			
	[106]					
∑ = 6	∑ = 13	∑ = 2	∑ = 12	∑ = 1	∑ = 0	∑ = 5

^m^The study uses a musculoskeletal model; ***°*** The applied control method considers multiple controller types and is therefore listed in more than one category

Frequently applied methods to describe postural control are P, PD and PID controllers. Depending on the controller type, P-, D- or I-terms are applied to minimize an error signal $e\left(t\right)$ (P-term), its derivative (D-term), or its integral (I-term) between a desired and an actual value of the kinematics or dynamics in a feedback loop. This way the control variable $u\left(t\right)$ can be adjusted. Equation (1) shows the general function of a PID controller:

1 $$u\left(t\right)={K}_{p}e\left(t\right)+{K}_{D}\frac{de\left(t\right)}{dt}+{K}_{I}\int e\left(t\right)dt ,$$

where ${K}_{p}, {K}_{D}$ and ${K}_{I}$ are gain constants of the P-, D- and I-terms and specify the magnitude of the different components [74]. When a PD controller is applied, the integral gain is zero, while for a P controller, both the integral and derivative gain are zero. In total, 69 times P, PD or PID controllers are applied to postural control models. P controllers are used 9 times, while PD controllers are considered 43 times, and PID controllers 17 times. Going more into detail, the distribution of the applied methods can be separated into models that simulate standing or gait. The P controller is used for 3 standing and 6 gait models. PD control is considered 30 times for standing simulations and 13 times for gait simulations. PID controllers are applied to 15 standing models and 2 gait model. Controlled variables in these models are joint angles, angular velocities [25, 44], the CoM [14, 47], or muscle length or muscle force when those are included in the (musculoskeletal) model [46].

Another approach we call *extended proportional (extended P) control *which is introduced in literature as “reflex-based control” [24]. Here, a musculoskeletal model is controlled by muscle reflexes. This type of model is based on muscle force and length feedback and can generate human walking dynamics without an additional feedforward mechanism. The main components of reflex control, the muscle force and length feedback, have an equation similar to a P controller. Since we aim to create a comparable level to the other control methods, we call this approach *extended P controller* here. Additionally, the model contains a PD control term for trunk control as a modulation of vestibular reflexes. Extended P controllers are applied 14 times, 13 of which are based on the first introduction of the model by Geyer & Herr [24]. 2 of these models are focusing on standing, the other 12 (including Geyer & Herr [24] are dealing with gait simulations. The modelled reflexes differ between muscles and, when simulating gait, between gait phases as well.

A linear quadratic regulator (LQR) can also be used to model the dynamic behaviour of human postural control. An LQR is a state-feedback controller where the feedback minimizes a quadratic cost function that penalizes control signals and state variables [53]. This controller requires linear mechanical system dynamics and a quadratic cost function [96]. Linear dynamics can be achieved for a human, who has nonlinear dynamics, during standing, when it is assumed that the range of motion is relatively small [96]. This kind of method is applied based on the hypothesis that the body tries to minimize a cost function [53]. A cost function can be achieved by assuming that the signals in the CNS are optimized through a quadratic function over the states and controls, which is valid since there is a redundant set of actuators and sensors in the human body which allows the CNS to pick an optimal combination of these [96]. An LQR is found in 9 postural control models that are contained in this review, 8 of them simulating human standing, 1 in combination with a P controller to simulate human gait [17].

Another method that can be applied to simulate postural control behaviour is model predictive control (MPC). This approach considers current input signals, output signals and predicted values and minimizes a cost function over a specific time horizon. It is an iterative process in discrete time which is repeated at each time step. MPC gives a numerical solution to an analytically unsolvable optimal control problem that works for nonlinear systems as well as linear systems [62]. This method is considered in 4 studies of this literature review, all of them focusing on standing.

Finally, 12 studies used methods that did not fit into the identified clusters. We found studies applying empirically derived equations for postural control feedback [31, 32, 55, 71, 101, 109], full-state feedback methods with additional accelerative information next to P- and D-terms [52, 104, 107], solely passive mechanisms without any active feedback [59, 105], or fuzzy controllers [40].

Figure 4 shows which postural control methods are used in combination with which biomechanical model type, separated into musculoskeletal and non-musculoskeletal human models as well as standing and gait simulations. In total 4 P, 13 PD and 3 PID controllers are applied to musculoskeletal models. Additionally, all 14 extended P controllers, as well as 6 of 12 methods that did not fall into a specific cluster, used musculoskeletal models, while none but 1 musculoskeletal model, that combines P control and an LQR, is used with one of the optimal control methods (LQR and MPC).

A topic discussed in literature is the influence of feedforward mechanisms to postural control and gait generation [29]. Central pattern generators for example could be a solution to generate a cyclic gait behaviour. Some of the authors use a combination of feedback and such feedforward elements to generate gait movements [19, 72, 73, 94]. However, a feedforward component is not necessary to create gait movements, as has been shown with the extended P controllers (e.g. Geyer & Herr [24]).

Another varying aspect within the different postural control models is the considered time delay describing afferences, efferences, processing time or parts of these. Included studies consider time delays that range from 0 ms [38, 59] up to 200 ms [7].

## Discussion

### Resolving the research questions

The systematic review revealed the following answers to our research questions:

RQ1: Various biomechanical human models are used to simulate postural control. During standing simulations mainly non-musculoskeletal models are applied and within these models mostly 1-joint models. When a musculoskeletal model is used during standing, there is no clear preferred model type. During gait simulations, mainly musculoskeletal models are applied, mostly 6-joint models considering left and right ankle, knee, and hip joints. When a non-musculoskeletal model is used for gait simulations, there is no clear preferred model type.

RQ2: The different human sensory systems are represented by tracking several types of information and internal states of the biomechanical human model, such as joint angles, global position, and orientation of specific segments in abstract biomechanical models, or muscle lengths, muscle velocities or muscle forces in musculoskeletal models. Additionally, ground reaction forces are considered as a representation of cutaneous receptors on the plantar surface of the feet. This way, different sensory systems of the body can be modelled for postural control. To fuse these different signals, sensory integration methods are implemented using summation of independent sensory channels, summation of sensory channels allowing for intersensory interactions or optimal estimator methods for sensory reweighing like Kalman filters. The independent channel model and the intersensory interaction model show to be the mainly applied methods when multiple sensory information is fused. Despite these different implementation methods, the underlying neural processes that exist in the human body are still unclear.

RQ3: Literature shows a variety of control methods to implement postural control mechanisms in biomechanical human models. Optimal control methods such as LQRs and MPCs are used as well as P, PD, PID and extended P controllers. For more complex biomechanical human models, with more DoF, the latter ones seem to be preferred to simulate postural control behaviour. PD and extended P controllers show to be the preferred controller types in musculoskeletal models. Whether other methods might provide promising results for physiologically-based simulations, meaning if it is possible to stabilize complex biomechanical human models using these methods, still needs to be investigated.

### Relation of biomechanical models and applied postural control methods

It is emerging that biomechanical human model types are related to postural control methods that are used and vice versa. PD controllers are used most often in general. However, for musculoskeletal models, especially for gait simulations of postural control, extended P controllers are predominant as well. In the 102 identified studies a total of 108 control approaches is applied that we clustered into 6 different types of control methods. Over all analysed studies, the PD controller is applied most (43 out of 108). All other approaches are distributed somewhat equally (9 P, 17 PID, 14 extended P, 9 LQR, 4 MPC, 12 other). This is the same when considering only standing models, while P controllers and extended P controllers are used the least. When considering gait models (in total 39), P controllers (6 times) and extended P controllers (12 times) are often used, while PD controllers are still chosen most (13 times). Optimal control methods (LQR and MPC) are not used for gait simulations except for one combination of a P controller with an LQR [17]. When a musculoskeletal model is chosen as biomechanical human model (in total 41 out of 108 times), PD (13 times) and extended P controllers (14 times) are chosen as control method most often, while they are never combined with LQRs or MPCs, neither for standing nor gait simulations except for one musculoskeletal gait model that combines a P controller with an LQR. PD (6 times) and extended P controllers (2 times) are predominant for musculoskeletal standing control (in total 11 models), while the extended P controller is the prime control method for musculoskeletal gait control (12 out of 30). An explanation for this shift from dominant PD controllers in general (and an equal distribution of the other methods) to mainly PD and extended P controllers for musculoskeletal models could be, that both perform well for models with a large number of DoF. At the same time, extended P controllers are the most applied control method for musculoskeletal gait models. The extended P controller requires muscle dynamics to be included in the model. It aims to describe human-like motion during postural control and implements reflexes with neurophysiological evidence [24]. It has to be mentioned, that extended P controllers are able to generate walking based solely on muscle reflexes, which might not be the only effector in reality. Moreover, in the models that are included in this literature review, LQRs as well as MPCs are rarely used for simulating model dynamics that are more complex, either by having a large number of DoF or when muscle dynamics are considered. Only one model represents at least a combination of a P controller and an LQR. This rare application might be due to the complexity of the models and the correspondingly large number of free parameters that have to be determined. Nevertheless, it is still reasonable that the human body considers an optimization problem to find a suitable reaction during postural control.

### Characteristics of sensory integration methods

As described in Sect. "Sensory representation and integration methods", different approaches exist to imitate the body’s sensory perception. When multiple sensory systems of the body are included, it is necessary to fuse this information using integration methods. Since underlying neural processes, meaning the processing of receptors’ information by the CNS, are still unknown [47, 107], studies describe different approaches for sensory integration. The several ways to simulate that process show to provide reasonable results for the specific application cases when simulation data is compared to experimental measurements. It is important to consider, even if simulation data are similar to experimental data, that the human’s processing could still be implemented differently in reality. Nevertheless, it is important that sensory systems are represented accurately when using postural control simulations for therapy recommendations in the future. Simpler methods, such as the summation of different receptors’ information (independent channel models) could show limitations in case of disturbances or absence of one or more sensor types. In these cases, the body had to reweigh the several input channels to ensure reliable estimates about its internal states [97, 98].

### Characteristics of different postural control models

Limitations and potentials of postural control models are mainly related to the way how a model simulates reactive behaviour of the biomechanical system. In the following, common characteristics as well as the different clusters identified in Sect. "Postural control methods" are discussed.

In general, most simulation data are compared to new or existing experimental measurement data to evaluate the simulation model. In each of the identified clusters for postural control methods simulation data often show human-like motion behaviour [3, 21, 24, 74, 77, 109]. But different comparison methods, like correlation of joint angles and joint movements [19, 56], muscle activations [19, 109], cross-correlation values [21] or gait cycle parameters [52], render a direct comparison difficult. It also remains difficult to assess whether models showing comparable movements to experimental data are realistically representing human’s internal processes or just generating human-like motion in a different way. For clinical applications, for example, it would be relevant not only to generate human-like motion behaviour, but to realistically represent the body’s internal processes. Additionally, many of the models have been linearized. This provides a simpler mechanical model, an easier implementation, and less unknown parameters, which then enables faster calculations. In contrast to this, nonlinearities of the human body (and motion) are ignored, and some characteristic dynamic behaviour will not be covered by these kinds of simulations, such as muscle behaviour.

Within the control methods, P, PD and PID controllers show different characteristics, but can all be implemented comparably easy [44]. They show several characteristics that can be utilized in human’s postural control simulations. The proportional gain of a P, PD or PID controller acts like a spring constant. When the gain is increased, the range of body sway decreases, and the frequency of body sway increases simultaneously in simulations of standing. By P control the reaction speed of a system can be controlled, but a P controller alone can lead to a steady difference between the desired and the actual value of the system output. That leads to a steady state error. At the same time the implementation is comparably simple as the proportional gain is the only parameter that has to be determined. The derivative gains of PD or PID controllers serve as damping constants. When these gains are increased the amplitude of body sway decreases, respectively [44]. For example, the corrective torque of a joint would be proportional to the velocity of body sway. Some authors found that velocity of movements provides sufficient information about changes of the CoM [65]. At the same time, PD control seems to show disadvantages if time delay is considered in the feedback loop [7, 53]. The integral gain of a PID controller shows to be relevant to keep the body’s sway oscillating around a specific reference point of a balanced position in simulations of standing. Otherwise the oscillation could occur around a deviated point [44]. Therefore the integral term controls the steady state error of the mechanical system [75]. Comparing PID and PD controllers, the body would oscillate about a set point when using PD control during quiet standing, while applying PID control can eliminate this oscillation [66]. It should be mentioned that some sway around a stable position typically appears in human standing.

In comparison to these kind of controllers, extended P controllers aim to create human-like motion considering some physiological processes like muscle reflexes. Haeufle et al. [30] found that a reflex-based model using muscle actuators requires less information compared to a torque-driven PD control model to generate the same movement, which seems plausible for human’s internal processes as well. Extended P controllers seem to be very promising using musculoskeletal models and gait simulations.

Models applying LQR can be implemented using few variables after simplification and linearization [60]. This way they represent a simple technique to design full-state feedback systems. By doing so, the number of gains has to be kept to a minimum [60]. Simulations can then be generated in a computationally efficient way. Model simulations deliver smooth and stable trajectories [96]. The linearization brings limitations due to necessary simplifications and assumptions compared to the real biomechanical system [76], even though differences between such linearized and nonlinear systems have never been examined in the included literature of this review. As soon as the models get more complex and passive structures should be involved as well, for example, a nonlinear control system is needed [97].

The postural control models based on MPC are characterized by a simple implementation and in the case of linearized models by computational efficiency [3]. MPC has advantages over LQR, since the biomechanical system requires less energy for the same motion task [3]. They also show for nonlinear problems to be quickly solvable and accurate by discretizing time [62].

All control methods discussed here have in common, that free parameters like control gains have to be found. To determine these gains, different strategies could be identified in the included studies. Some of the authors use hand-tuned or literature-based parameters to describe the controller’s equations [24, 65], others apply different optimizing algorithms to find optimal values for control parameters [60, 88]. The choice of different optimization methods can depend on the optimization goal. For example, it may be minimizing the deviation from an existing measurement [52] or predefined reference pose [38], or mimicking human physiological processes. In the latter case, an assumption can be the body’s optimization of its energy consumption [83].

### Limitations of the study

In the context of postural control simulations, authors focus on many different fields that are part of this research topic. Some authors try to determine specific parameters from experimental data, other authors investigate the influence of special tasks on postural control and others build models that can be used for forward dynamic simulations. Therefore, it is difficult to find a compromise between general and specific search strategy when a systematic literature research is performed by applying a search string. The nomenclature of postural control is not consistent in literature too, as already mentioned in Sect. "Biological background". That is why the search string served as a starting point and was combined with an additional backward search. Some studies do not describe the control models in detail, and instead focus on other areas of the simulation process. Therefore, these studies are not included in the classification. Many models are used to investigate the principles of postural control but are applied to robotic systems like humanoids or exoskeletons, to special motion tasks or to persons with specific diseases like post-stroke patients. These special cases are also not considered in this review, as we want to give an overview about human postural control models on a comparable level of healthy standing and gait.

## Conclusion and outlook

We conducted a systematic literature review to get an overview of different approaches used to simulate postural control in biomechanical human models as we aim to use postural control simulations to give therapy recommendations to patients suffering from postural disorders in the future. This review aims to give a general overview and impression about the various methods that have been published to simulate the human’s postural control behaviour. The process of postural control consists of the sensory perception of the internal states and dynamics of the body, the sensory integration of these different types of sensory information and a control mechanism that determines which reactions should be executed based on this considered information. In literature various models exist that include different sensory systems observed by specific receptors, sensory integration methods and control strategies to describe and imitate human’s postural control behaviour. The choice of these depends on the level of detail in the model and the scope of the study. Many methods show movements comparable to human behaviour, but it is still unknown exactly which processes take place in the actual human’s CNS to process its sensory information. Nevertheless, it can be recognized that the more detailed the biomechanical human models are, and the more complex the observed motion, increasingly extended P controllers are applied to describe the human’s postural control.

Many models in this literature research focus on simple methods to simulate the general movement characteristics (RQ1), others focus on sensory integration mechanisms and consider more than one sensory system using a simplified biomechanical model (RQ2). Some authors use musculoskeletal models focusing on one sensory system. We found only few studies considering more than one human sensory system for postural control. These are first promising approaches to model the human’s postural control system with a detailed biomechanical human model including multiple sensory systems. To control musculoskeletal models in general, mainly PD and extended P controllers are applied, while to control musculoskeletal models during gait simulations, mainly extended P controllers are applied (RQ3). Whether these are applied for more complex human models because other control methods could not be stabilized and whether other control models would still be promising to simulate postural control, is unclear and could be investigated in the future.

Different comparison methods of simulation outcomes with experimental measurement data render a direct comparison of the accuracy of different postural control models difficult. Therefore, it would be interesting for future research to directly compare different existing postural control models.

Another promising area of research and our purpose for this review is simulating postural control behaviour of humans, in standing and gait, both from healthy as well as people with motor impairments. Doing so, postural control of people, whose motor control is affected by disease, can be simulated and compared to healthy control behaviour. This approach can support therapy recommendations, for example to suggest targeted exercises to improve mobility, stability, and responsiveness. This review helps to assess which methods already exist and which of them show to be promising for simulating postural control on a physiologically-based way.

## Data Availability

All data generated or analysed during this study are available from the corresponding author on reasonable request.

## Abbreviations

CNS: Central nervous system
CoM: Centre of mass
DoF: Degree of freedom
LQR: Linear quadratic regulator
MPC: Model predictive control
P controller: Proportional controller
PD controller: Proportional-derivative controller
PID controller: Proportional-integral-derivative controller
